# Whole-Genome Sequencing Elucidates the Epidemiology of Multidrug-Resistant *Acinetobacter baumannii* in an Intensive Care Unit

**DOI:** 10.3389/fmicb.2021.715568

**Published:** 2021-09-13

**Authors:** Pu Mao, Xiaolong Deng, Leping Yan, Ya Wang, Yueting Jiang, Rong Zhang, Chun Yang, Yonghao Xu, Xiaoqing Liu, Yimin Li

**Affiliations:** State Key Laboratory of Respiratory Diseases, National Clinical Research Center for Respiratory Disease, Guangzhou Institute of Respiratory Health, The First Affiliated Hospital of Guangzhou Medical University, Guangzhou, China

**Keywords:** *Acinetobacter baumannii*, epidemiology, whole-genome sequencing, intensive care unit, multi-drug resistance

## Abstract

The nosocomial pathogen *Acinetobacter baumannii* is a frequent cause of healthcare-acquired infections, particularly in critically ill patients, and is of serious concern due to its potential for acquired multidrug resistance. Whole-genome sequencing (WGS) is increasingly used to obtain a high-resolution view of relationships between isolates, which helps in controlling healthcare-acquired infections. Here, we conducted a retrospective study to identify epidemic situations and assess the percentage of transmission in intensive care units (ICUs). Multidrug-resistant *A. baumannii* (MDR-AB) were continuously isolated from the lower respiratory tract of different patients (at the first isolation in our ICU). We performed WGS, pulsed-field gel electrophoresis (PFGE), and multilocus-sequence typing (MLST) analyses to elucidate bacterial relatedness and to compare the performance of conventional methods with WGS for typing MDR-AB. From June 2017 to August 2018, *A. baumannii* complex strains were detected in 124 of 796 patients during their ICU stays, 103 of which were MDR-AB. Then we subjected 70 available MDR-AB strains to typing with WGS, PFGE, and MLST. Among the 70 *A. baumannii* isolates, 38 (54.29%) were isolated at admission, and 32(45.71%) were acquisition isolates. MLST identified 12 unique sequence types, a novel ST (ST2367) was founded. PFGE revealed 16 different pulsotypes. Finally, 38 genotypes and 23 transmissions were identified by WGS. Transmission was the main mode of MDR-AB acquisition in our ICU. Our results demonstrated that WGS was a discriminatory technique for epidemiological healthcare-infection studies. The technique should greatly benefit the identification of epidemic situations and controlling transmission events in the near future.

## Introduction

*Acinetobacter baumannii* is a Gram-negative pathogen that causes serious nosocomial infections, especially in patients with advanced age, mechanical ventilation, respiratory failure, or a prolonged hospital stay in intensive care units (ICUs) (Vazquez-Lopez et al., 2020). In recent decades, *A. baumannii* has emerged as an important nosocomial pathogen that exhibits high levels of resistance to antibiotics and has become endemic in some geographical regions. Multidrug-resistant *A. baumannii* (MDR-AB) increases hospital stays and costs and limits therapeutic options. Critically ill patients infected with MDR-AB strains have substantially higher mortality rates (Falagas et al., 2006).

Accurate strain classification is a key process necessary for managing antimicrobial-resistant strains in health care-related infections (Kalenic and Budimir, 2009; Nishi and Hidaka, 2016). Pulsed-field gel electrophoresis (PFGE) is still used for typing analysis of *A. baumannii*, although it has drawbacks in terms of the time requirement and difficulty in data interpretation (Thong et al., 2002; Singh et al., 2006). Multilocus-sequence typing (MLST), the partial sequence-based typing method, is usually considered as the gold standard for global epidemiological investigations. It focuses on the clustering of isolates worldwide and is not sensitive to short duration and small-scale outbreaks (Tomaschek et al., 2016). It is considered a complementary approach of PFGE since it connects isolates with global epidemiology (Tomaschek et al., 2016). In a decade, whole-genome sequencing (WGS) has become a promising method in microbiological laboratories for strain identification, molecular epidemiology and outbreak analysis (Makke et al., 2020). It has the highest discriminatory power and is likely to be widely used with cost reduction (Halachev et al., 2014; Salipante et al., 2015; Hwang et al., 2021).

In this study, we conducted a retrospective study to identify an epidemic situation and MDR-AB-transmission events. We used reference-based single-nucleotide polymorphism (SNP) approaches to map either processed reads or assembled contigs to a reference genome, followed by SNPs calling. Then, SNPs differences among the MDR-AB isolates were processed. We aimed to describe the prevalence and molecular epidemiology of MDR-AB in the ICU through WGS-based typing and compare the performance of conventional methods with WGS for typing MDR-AB.

## Materials and Methods

### Setting

The First Affiliated Hospital of Guangzhou Medical University is a 1500-bed teaching hospital and a tertiary referral center for major respiratory disease in southern China. This study was conducted in the 27-bed adult ICU, which cares for patients with respiratory disease and those who have undergone thoracic surgical procedures. The ICU included 4 senior intensivists, 14 attendings, 6 residents, medical students, and nurses during the study period. Attendings and residents staffed the ICU 24 h per day and every day of the week. Three teams provided ICU medical coverage during the day: each including a senior intensivist, four attendings and two residents taking care of seven bed patients. The nurse-to-patient ratio was maintained at 1–1.5:1 during daytime and 1:3 at night.

Infection control measures mainly implemented in ICU have been previously described (Ye et al., 2015), mainly included: performing hygiene according to the WHO recommended and re-emphasis on the appropriate use of gloves, especially not allowed wear gloves to contract clean area items; implementing a cohort strategy, colonized/infected patients were admitted to a special room and treated by a dedicated team of healthcare workers; practice contact precautions for colonized/infected and newly admitted patients. Artificial airway management followed Critical Care Medicine Branch of Chinese Medical Association Guideline for diagnosis, prevention, and treatment of ventilator-associated pneumonia (Chinese Society of Critical Care Medicine, 2013).

Ethical approval was not required because the study was conducted as part of normal surveillance and management of healthcare-associated infections.

### Bacterial Identification and Antimicrobial-Susceptibility Testing

Seventy-five MDR-AB complex isolates were collected from the lower respiratory tract of hospitalized ICU patients between June 2017 and August 2018 and were designated AB-1 to AB-75, based on admission date. The lower respiratory tract specimens were obtained by tracheal aspiration.

Strains were cryopreserved until needed for WGS. Initial identification was conducted using the VITEK2 Compact 30 System (bioMérieux). Antimicrobial susceptibility was determined *via* microdilution in accordance with guidelines of the Clinical and Laboratory Standards Institute (M100-S29). MDR was defined as resistance to at least one representative agent from three or more of the five antimicrobial-agent classes: cephalosporins (such as ceftazidime or cefepime), carbapenems (such as imipenem), β-lactamase inhibitors (such as cefoperazone/sulbactam), fluoroquinolones (such as ciprofloxacin), and aminoglycosides (such as amikacin) (Magiorakos et al., 2012).

### PFGE Analysis

Isolates were inoculated in liquid Luria-Bertani medium and cultured overnight at 37°C. The bacterial supernatant was collected through centrifugation and resuspended in 1× Tris-ethylenediaminetetraacetic acid (EDTA) buffer (TE buffer) before an equal volume of 2% clean cut agarose was added. After immediate mixing, the liquid was placed in a mold and allowed to solidify. The block was then placed into 300 μL cell lysate (in 100 mmol/L Tris and 100 mmol/L EDTA), mixed with 5 μL proteinase K (20 mg/mL), and incubated overnight at 50°C. Next, the block was washed three times with 1 × TE, for 1 h/wash step, before being incubated overnight at 25°C with 50 U Apa I enzyme. Subsequently, PFGE was performed using a CHEF-DR III PFGE apparatus, under the following conditions: running buffer, 0.5 × TBE; 1% Pulsed-Field Certified Agarose; 14°C; 6 V/cm; angle of 120°; 0.5–20 s, overall time: 20 h. After the current ended, ethidium bromide staining was performed and images were obtained. Fingerprints were analyzed by BioNumerics software, using the unweighted pair group method and the arithmetic averages method, a 2% tolerance in strip position difference, and a 0.8% optimization value. Strains with ≥87% similarity were classified as the same subtype (representing the same clone), and those with <87% similarity were classified as different genotypes (representing different clones) (Seifert et al., 2005).

### WGS and Assembly

Genomic DNA was extracted from 3 mL overnight cultures of 75 *A. baumannii* complex isolates, using the SPARK DNA Sample Prep Kit-96 (Enzymatics, United States). A library was generated for each isolate using the NEB Next Ultra DNA Library Prep Kit (Illumina, San Diego, CA, United States). DNA library concentrations were quantified using a Qubit^®^ 2.0 Fluorometer and a Qubit^TM^ dsDNA HS Assay Kit, and 150-base pair (bp) paired-end reads were generated with a HiSeq^®^2500 instrument. Sequencing was performed at the Shanghai Biotechnology Corporation. We implemented a filtering pipeline that trimmed reads from the 3′ end with <20 sequencing quality, discarded adaptor sequences and reads <45 bp in length, removed reads containing Ns or where <50% of bases had >20 sequencing quality. Assembly statistics are displayed in Supplementary Table S1.

### MLST Analysis

Conventional MLSTs were inferred *in silico* from WGS data. Seven MLST loci were selected using a sequence-extraction tool and then identified using a public *A. baumannii* MLST database.^1^

### Single-Nucleotide Polymorphism Detection and Whole-Genome Phylogeny

Paired reads were mapped to *A. baumannii* strain XH386 (reference genome; GenBank accession number NZ_CP010779.1) using the Burrows–Wheeler Aligner (version 0.7.12). The original file was converted to a BAM file in SamTools. SNP and InDel results were processed simultaneously using the HaplotypeCaller tool, based on a real-time *de novo* algorithm with BAM files (reserved mutation sites with sequencing quality >30) and obtained VCF files. The SNPhylo program was used to remove low-quality SNPs; processed SNPs were then concatenated into a sequence and compared in the MUSCLE program. During this process, we discarded the following single-nucleotide variations (SNVs): (1) those in genes annotated as phages, transposases, or integrases, (2) those in genomic regions annotated as phages using Phage Finder, (3) those within 20 bp of the start or end of a contig, and (4) those with sequence quality <100. Tree construction was performed in PHYLIP. Trees were visualized in Evolview version 3.0.^2^ This analysis was performed at the Shanghai Biotechnology Corporation.

Previously, *A. baumannii* was estimated to have a ∼5 SNPs/year mutation rate over the whole genome (Hawkey et al., 2018). Isolates were assigned as the same genotype if their SNV numbers were ≤10.

### Nucleotide Sequence Accession Numbers

Generated sequence reads were submitted to the NCBI Sequence Read Archive under the accession number PRJNA639298.

## Results

### Bacterial Isolates and Patients

This study spanned the period from June 2017 to August 2018, when 868 admissions (involving 796 patients) to the ICU were recorded. After deleting repetitive samples from the same patients with same isolates, we identified 124 samples as *A. baumannii* complex or *A. baumannii* using the VITEK2 Compact 30 System, of which 103 showed MDR.

Among those 103 samples, the first 75 MDR-AB complex and *A. baumannii* isolates obtained were retrieved from storage; WGS successfully analyzed 71 isolates. Seventy isolates were determined to be *A. baumannii*, and one isolate was identified as *Acinetobacter calcoaceticus*.

The 70 MDR-AB strains analyzed using WGS were from 70 different patients. The clinical characteristics of these 70 patients are summarized in Table 1.

**TABLE 1:** Clinical characteristics of the patients with MDR-AB sequenced in this study.

Characteristics^a^	
Age (years)	60.09 ± 17.01
Gender, male	45 (64.29)
Prior hospital stay (<1 year)	17 (24.29)
Location before ICU admission	
Internal^b^	35 (50.00)
Outside^c^	35 (50.00)
Days of stay in ICU	28.50 (11.75–48.75)
Days of ventilation	31.00 (18.75–66.00)
Days of ICU stay after MDR-AB isolated	22.50 (9.00–43.25)
APACHE II score upon ICU admission	18.71 ± 6.57
SOFA score upon ICU admission	8.66 ± 4.12
Primary ICU admission diagnosis	
Chronic pulmonary disease	25 (35.71)
Pneumonia	44 (62.85)
Diabetes mellitus	18 (25.71)
Hypertension	24 (34.29)
Malignancy	16 (22.86)
Surgery procedure (< 30 days)	17 (24.29)
Antibiotics used before ICU admission	66 (94.29)
Antibiotic class^d^	
Carbapenem	45 (64.29)
Penicillin	1 (1.43)
Piperacillin–tazobactam	42 (60.00)
Cephalosporin	17 (24.29)
Fluoroquinolones	23 (32.90)
Glycopeptide	26 (37.14)
Antifungal	43 (61.43)
Invasive procedures	
Ventilator	67 (95.71)
Blood transfusion	7 (10.00)
Hemodialysis	7 (10.00)
Nasogastric tube	2 (2.86)
Peripheral venous catheter	5 (7.14)
Central venous catheter	60 (85.71)
Urinary catheter	64 (91.43)
Outcome	
28-day mortality	3 (4.29)
Hospital mortality	17 (24.29)

*^*a*^All data are presented as the number, with the percentage in parenthesis, except for the age, APACHE II score, and SOFA score, which are presented as the mean ± SD. The number of days in the ICU, the number of days of ventilation, and the number of days in the ICU after MDR-AB was isolated are presented as median (IQR).*

*^*b*^Internal, patients were transferred from other departments in our hospital.*

*^*c*^Outside, patients transferred from the other hospitals.*

*^*d*^Use of antibiotics in patients: used within 30 days before the first MDR-AB isolate was discovered and the antibiotics were used for at least 72 h.*

### Presentation With MDR-AB Upon ICU Admission and MDR-AB Acquisition in the ICU

All patients were screened for lower-respiratory tract infections within 24 h of admission and assessed at least once/week. Acquisition with MDR-AB was defined by a change in the colonization status from culture-negative to positive or a change of strains based on epidemiological data. During the study period, 55 patients (53.4%, 55/103) carried MDR-AB at the time of admission, and 48 acquisitions were identified. Among the 70 patients whose isolates were analyzed with WGS, 54.28% (*n* = 38) carried MDR-AB at the time of admission. Thirty-two acquisitions were identified: 13 patients who were negative during the first screening showed MDR-AB in a later screening; 19 patients showed changes of MDR-AB strains.

### Antibiotic-Susceptibility Testing

Drug-sensitivity test results of the 70 MDR-AB strains are shown in Supplementary Table S2. Almost all isolates were resistant to quinolones. We found that 100% of the MDR-AB strains (*n* = 70) were resistant to ciprofloxacin, 80% (*n* = 56) were resistant to levofloxacin, and 14 isolates showed intermediate resistance to levofloxacin. Resistance rates to third-generation cephalosporins (cefotaxime and ceftazidime) were 91.43 and 97.14%, respectively. In addition, 98.57% (*n* = 69) were resistant to imipenem or piperacillin–tazobactam, 97.14% (*n* = 68) were resistant to meropenem, and all tested isolates were sensitive to colistin (six strains were untested).

### MLST Analysis

**FIGURE 1:**
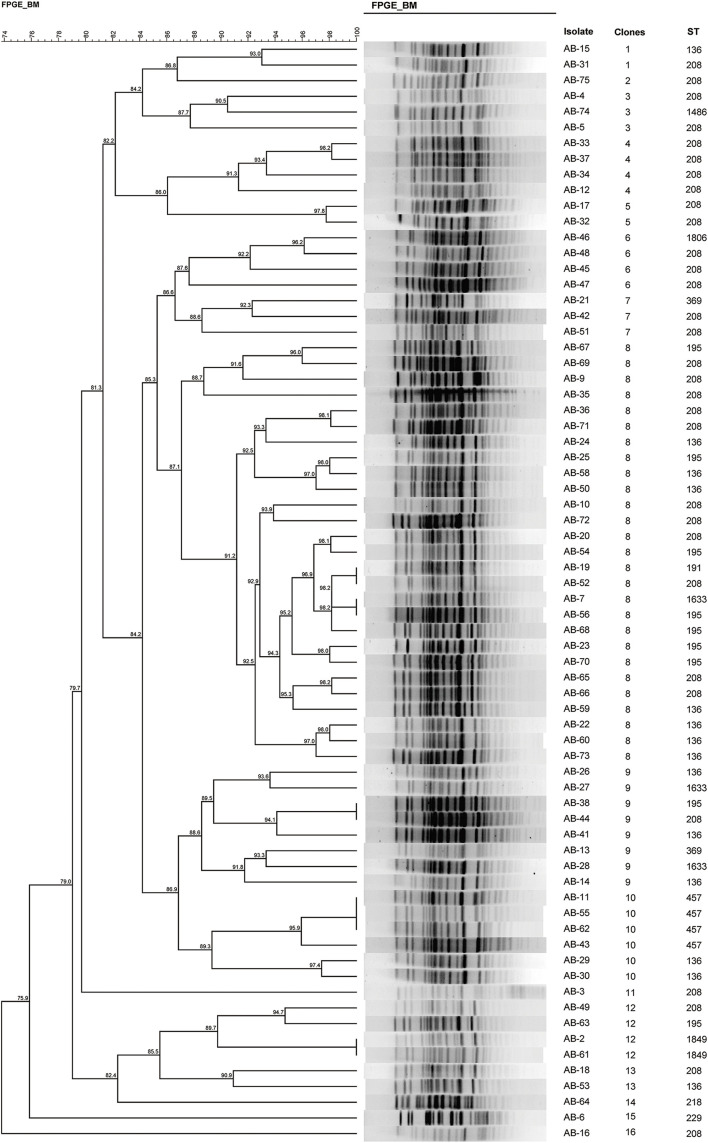
Pulsed-field gel electrophoresis profiles of MDR-AB and dendrogram, and MLST.

Ten different STs were identified using the Oxford scheme, whereas only two STs were identified using the Pasteur MLST scheme. Considering that the Oxford scheme shows a higher concordance with WGS phylogenies and better discriminates between strains with short evolutionary distances, we adopted the Oxford scheme for further analysis. We identified 12 unique STs among the 70 *A. baumannii* isolates, namely ST208 (*n* = 31), ST136 (*n* = 14), ST195 (*n* = 9), ST457 (*n* = 4), ST1633 (*n* = 3), ST1849 (*n* = 2), ST369 (*n* = 2), ST229 (*n* = 1), ST191 (*n* = 1), ST1486 (*n* = 1), ST1806 (*n* = 1), and a novel ST, ST2367(*n* = 1) (Figure 1). Except for the ST229 clone, belonging to complex 110 international clone VII (CC110 IC7), the remaining isolates were assigned to clonal complex 92 (also known as international clone II), widely epidemic in over 30 countries (Karah et al., 2012; Tomaschek et al., 2016). Our study identified three major STs, with the predominant population being ST208, accounting for 44.29% (31/70). ST136 was the second-most prevalent clone (20.00%, 14/70), followed by ST195 (12.86%, 9/70).

### PFGE Analysis

During the period when these strains were collected, we also typed isolates using PFGE. Strains with ≥87% similarity were considered the same subtype, representing the same clone (Seifert et al., 2005). Accordingly, we identified 16 different pulsotypes (Figure 1). Notably, 27 strains were classified as clone 8, making it the most abundant epidemic clone, and it continued to spread through the ICU during the study period.

### Whole-Genome SNP Phylogenetic Analysis

**FIGURE 2:**
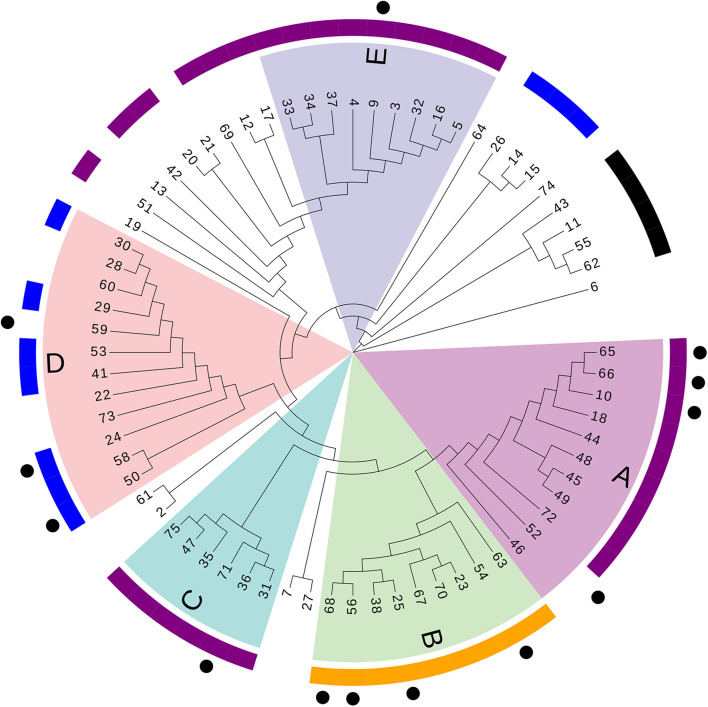
Phylogenetic tree based on SNPs. Numbers refer to the ID number of isolates. Different letters (A–E) and colors refer to branches of the five major clades. Outer purple ring, ST208; outer yellow ring, ST195; outer blue ring, ST136; outer black ring, ST457. Dots represent acquisitions of MDR-AB isolates of clone 8 with the same PFGE type.

A phylogenetic tree based on SNPs was constructed to clarify relationships among the sequenced strains. The median of the minimum SNV number across different isolates during the research period was 426 (interquartile range: 275–948; range: 0–52,781). Five distinct primary clades were identified (clades A–E; Figure 2).

**FIGURE 3:**
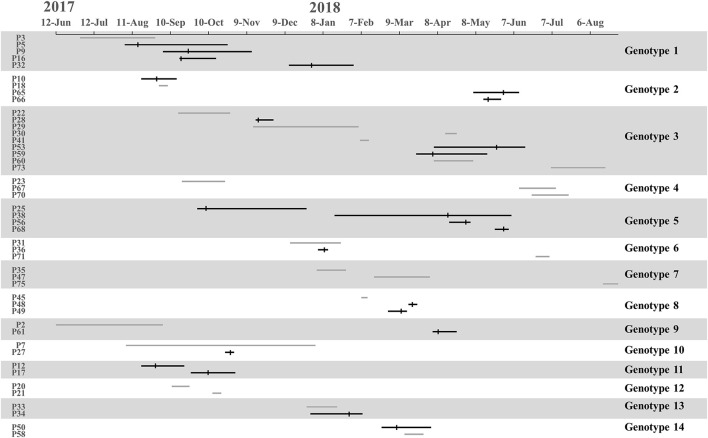
Timelines and overlap of major MDR-AB genotypes. The line indicates stay periods in the ICU. The gray line represents patients in whom MDR-AB isolates were detected before ICU admission. Vertical bars indicate the time at which MDR-AB-positive samples were detected.

We found 38 genotypes, with 14 genotypes identified in more than one patient. Along the 14 genotypes identified in more than one patient, we found that five genotypes consist of 16 instances where patients carried MDR-AB at the time of admission, suggesting that a common source of infection existed outside of the ICU (Figure 3).

Transmission was defined as patient acquisition of a genotype cultured from a previous patient, irrespective of overlapping patient stay in the ICU. Under WGS, transmission occurred when SNVs ≤10 between isolated pathogens. We identified 23 transmissions for 11 genotypes, consisting of 16 transmissions where donors were patients who carried MDR-AB at the time of admission (for eight genotypes), and 7 transmissions where the donor was not identified (for three genotypes) (Figure 3). Genotypes 1 and 5 showed the highest number of transmissions (four cases in each genotype). These results demonstrate that the major source of transmissions in our ICU was the admission isolates, also suggested that MDR-AB spread in our ICU mainly through transmission.

**FIGURE 4:**
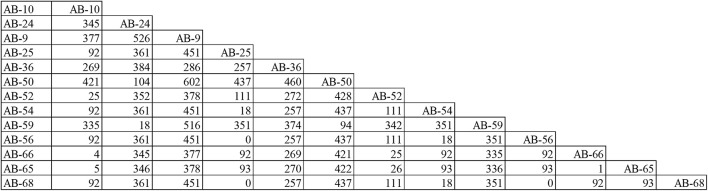
Single-nucleotide polymorphisms matrices for the 13 MDR-AB acquisitions belong to clone-8, as typed by PFGE.

According to PFGE results, 27 MDR-AB strains formed clone 8, the largest epidemic clone, which was associated with 13 acquisitions. We reexamined this prevalent clone using WGS to determine whether “outbreak” isolates and transmissions identified with this technique actually reflect the relationship between different pathogens. The 13 acquisitions of MDR-AB isolates belonging to clone 8 were divided into five WGS clades, involving seven genotypes (Figures 2, 3). Mean number of SNVs among them was 279, ranging from 0 to 602 (92–377; Figure 4), and six isolates had <10 SNVs.

## Discussion

In this study, we used WGS to retrospectively investigate the epidemiology of clinical MDR-AB isolates in an adult ICU and to determine how frequently MDR-AB is transmitted between ICU patients.

Data from a previous epidemiological WGS study of *Staphylococcus aureus* suggested that, under standard infection-control measures, transmissions contributed to a small part of *S. aureus* acquisitions (Price et al., 2014). Price et al. (2014) reported that just 14% of patients were potentially involved in transmission at an adult ICU and high-dependency unit. Our findings demonstrated that, among the 868 ICU admissions, 23 of 103 patients with lower-respiratory tract infections acquired MDR-AB through transmission. Several factors may have contributed to the higher frequency of transmission in our ICU. First, the primary diagnosis upon ICU admission was mainly pneumonia (44/70, 62.86%). In 27 of these patients MDR-AB were cultured. Second, the samples collected also different from those of previous studies, as diagnosing lower-respiratory tract infections was the main objective of our study. Third, although the number of beds in our ICU and the study duration were similar, length of stay in our ICU was significantly longer than others reported.

Since our hospital is a center of respiratory disease in southern China, one of the most common reasons for admission to ICU is critical patients cause by respiratory diseases. Half of the patients were transferred from other hospitals where they had been treated, and 38/70 patients (56.52%) carried MDR-AB at admission. Preventing the spread of MDR-AB was a big challenge for our ICU. In our study, only two patients did not require ventilation, and most patients used antibiotics before admission into the ICU. Mechanical ventilation frequently requires tracheal intubation, which increases the risk of inhaling pathogenic bacteria or gastrointestinal tract bacteria (Coffin et al., 2008; De Waele et al., 2010). Prior use of antibiotics was demonstrated as an independent risk factor for colonization/infections with multi-resistant Gram-negative bacteria (Vasudevan et al., 2013). Along the ICU patients, bronchoscopy is a common diagnostic and therapeutic procedure which could cause nosocomial outbreak (McGrath et al., 2017). Those factors all pose risks for acquisition of MDR-AB and may contribute to transmission.

Among the 38 admission isolates, 16 MDR-AB isolates were highly related (SNV <10) and belonged to four genotypes, comprising seven isolates of patients transferred from another hospital and nine isolates from a different department in our hospital. These findings suggest that MDR-AB had disseminated in our hospital and spread widely throughout the local Guangzhou area. The data also imply that MDR-AB adapted for persistence and transmission in a hospital environment.

*Acinetobacter baumannii* can survive in the environment for a long time and is potentially transmissible (Marchaim et al., 2007). Contaminated environments and equipment can act as reservoirs for MDR-AB (Chapartegui-Gonzalez et al., 2018). In our study, strains belonging to the same genotype isolated from different patients (such as genotype 1 and 5) were sustained for a long period, and the patients did not share the same time in the ICU. This indicated that some transmission events might have occurred indirectly *via* the contaminated environment or healthcare workers. These findings are consistent with previous research conducted in our ICU (Ye et al., 2015). Except for genotype 1 and 5, the remain genotypes involved in more than one patient did not persist for a longer time. In our study period, a bundle of infection-prevention measures was implemented, including hand hygiene, contact precautions, and cohorting with dedicated healthcare staff. All of the components of the infection prevention bundle played a role in controlling MDR-AB dissemination. Cohorting with dedicated healthcare staff was firstly implemented in 2015 in our ICU to control MDR-AB outbreak (Ye et al., 2015) and is still implemented up to now. This measure was recommended by the Centers for Disease Control (WHO, 2017) and Prevention and the European Centre for Disease Prevention and Control (Magiorakos et al., 2017) and was shown to be efficient (Abboud et al., 2016; Perez-Blanco et al., 2018). We suggested that cohort strategy maybe contribute to reduce the MDR-AB dissemination in our ICU.

Classifying pathogens to elucidate epidemiology of pathogenic bacteria and hospital outbreaks relies on typing techniques with higher discriminatory power. Using conventional epidemiological data, our study identified 32 potential acquisitions that involved 29 clones from more than two isolates identified through PFGE. When WGS data were included, we reduced the number of transmissions to 23. In comparison with PFGE ability to type MDR-AB, WGS separated isolates belonging to clone 8 into five different clades. Then, we examined the typing ability of MLST. Although each clade typed by WGS corresponded to one of the three predominant STs, ST208 was interspersed into three different clades. PFGE and MLST both show poor agreement with WGS. Our data indicated that WGS offers the advantage of resolving differences between closely related isolates (Zarrilli et al., 2013). Therefore, WGS was more suitable for typing and identifying transmission between patients, especially when the same pandemic ST isolates are identified regionally.

The PubMLST database assigned all strains to 12 STs that could be grouped into CC92 clonal complexes (also known as international clone II), except for ST229. The majority of isolates belonged to ST-208, which had spread throughout other provinces in China (Jiang et al., 2016; Ning et al., 2017). Between 2012 and 2015, ST457 was reported as a prevalent pathogen with enhanced virulence in five hospitals in southern China (Zhou et al., 2018). Our study identified just four strains belonging to ST457, three of which were isolated at the time of admission, and they were not closely related (>25 SNPs). These findings suggest our ICU was not associated with the new emerging clones.

This study had two major limitations. First, we did not test any environmental samples or healthcare workers’ hands, sources that may have contributed to the spread of MDR-AB. Second, considering the sensitivity of bacterial culture, some changes in colonization status from culture-negative to culture-positive may have been false-positives. Hence, rate of acquisition may have been overestimated.

## Conclusion

In conclusion, transmission mainly contributed to MDR-AB acquisition in our ICU; thus, prevention and control of MDR-AB hospital infections must be strengthened. Our study provides a high-resolution genome-wide perspective on MDR-AB epidemiology in a healthcare setting, while contributing to the development of appropriate intervention and prevention strategies.

## Data Availability Statement

The datasets presented in this study can be found in online repositories. The names of the repository/repositories and accession number(s) can be found in the article/Supplementary Material.

## Author Contributions

PM and XD participated in data analysis and drafted the manuscript. LY and YW carried out the molecular genetic studies. YJ participated in the clinical sample isolation and antibiotic testing. RZ and CY managed the data collection. YX, XL, and YL participated in the study design. All authors read and approved the final manuscript.

## Conflict of Interest

The authors declare that the research was conducted in the absence of any commercial or financial relationships that could be construed as a potential conflict of interest.

## Publisher’s Note

All claims expressed in this article are solely those of the authors and do not necessarily represent those of their affiliated organizations, or those of the publisher, the editors and the reviewers. Any product that may be evaluated in this article, or claim that may be made by its manufacturer, is not guaranteed or endorsed by the publisher.

## Footnotes

1
https://pubmlst.org/organisms/acinetobacter-baumannii

2
https://evolgenius.info/

## Supplementary Material

Supplementary Table S1 Whole-genome sequencing and assembly statistics of 70 isolates.

Supplementary Table S2 General patient information and the results of drug-sensitivity testing.
